# Scaling Up Early Infant Male Circumcision: Lessons From the Kingdom of Swaziland

**DOI:** 10.9745/GHSP-D-15-00186

**Published:** 2016-07-02

**Authors:** Laura Fitzgerald, Wendy Benzerga, Munamato Mirira, Tigistu Adamu, Tracey Shissler, Raymond Bitchong, Mandla Malaza, Makhosini Mamba, Paul Mangara, Kelly Curran, Thembisile Khumalo, Phumzile Mlambo, Emmanuel Njeuhmeli, Vusi Maziya

**Affiliations:** a Jhpiego, Baltimore, MD, USA; b U.S. Agency for International Development (USAID), Mbabane, Swaziland; c Raleigh Fitkin Memorial Hospital, Manzini, Swaziland; d Population Services International, Mbabane, Swaziland; e United Nations Children’s Fund (UNICEF), Mbabane, Swaziland; f Family Life Association of Swaziland, Manzini, Swaziland; g Swaziland Ministry of Health, Mbabane, Swaziland; h USAID, Washington, DC, USA; i Swaziland National AIDS Program, Mbabane, Swaziland

## Abstract

**Background:**

The government of the Kingdom of Swaziland recognizes that it must urgently scale up HIV prevention interventions, such as voluntary medical male circumcision (VMMC). Swaziland has adopted a 2-phase approach to male circumcision scale-up. The catch-up phase prioritizes VMMC services for adolescents and adults, while the sustainability phase involves the establishment of early infant male circumcision (EIMC). Swaziland does not have a modern-day tradition of circumcision, and the VMMC program has met with client demand challenges. However, since the launch of the EIMC program in 2010, Swaziland now leads the Eastern and Southern Africa region in the scale-up of EIMC. Here we review Swaziland’s program and its successes and challenges.

**Methods:**

From February to May 2014, we collected data while preparing Swaziland’s “Male Circumcision Strategic and Operational Plan for HIV Prevention 2014–2018.” We conducted structured stakeholder focus group discussions and in-depth interviews, and we collected EIMC service delivery data from an implementing partner responsible for VMMC and EIMC service delivery. Data were summarized in consolidated narratives.

**Results:**

Between 2010 and 2014, trained providers performed more than 5,000 EIMCs in 11 health care facilities in Swaziland, and they reported no moderate or severe adverse events. According to a broad group of EIMC program stakeholders, an EIMC program needs robust support from facility, regional, and national leadership, both within and outside of HIV prevention coordination bodies, to promote institutionalization and ownership. Providers and health care managers in 3 of Swaziland’s 4 regional hospitals suggest that when EIMC is introduced into reproductive, maternal, newborn, and child health platforms, dedicated staff attention can help ensure that EIMC is performed amid competing priorities. Creating informed demand from communities also supports EIMC as a service delivery priority. Formative research shows that EIMC programs should address the fears and anxieties of parents so that they, especially fathers, understand the health benefits of EIMC before the birth of their babies.

**Conclusion:**

The vast majority of public-sector facilities in Swaziland are led by nurses, and nurses and midwives have borne the brunt of caring for patients with HIV/AIDS in Swaziland. Like prevention of mother-to-child transmission, EIMC provides an opportunity for nurses and midwives to stand at the forefront of HIV prevention efforts. Rapid scale-up of VMMC and EIMC in Swaziland has the potential to avert more than 56,000 HIV infections and save US$370 million in the next 20 years.

## Abstract (precis)

Swaziland is the first country to introduce national early infant male circumcision (EIMC) into voluntary medical male circumcision (VMMC) programming for HIV prevention. With more than 5,000 EIMCs performed between 2010 and 2014, Swaziland learned that EIMC requires inclusion of stakeholders within and outside of HIV prevention bodies; robust support at the facility, regional, and national levels; and informed demand. Expansion of EIMC and VMMC has the potential to avert more than 56,000 HIV infections in Swaziland over the next 20 years.

## BACKGROUND

With an HIV prevalence of 26%^1^ among adults and 41.1%^2^ among pregnant women, the Kingdom of Swaziland faces a substantial HIV and AIDS burden. The government recognizes that in order to alleviate this burden, it must urgently scale up effective, evidence-based HIV prevention interventions. To contribute to this goal, the government prioritized voluntary medical male circumcision (VMMC) in its initial “National Strategic Framework for HIV and AIDS 2009–2014” because VMMC is a safe procedure that has reduced the risk of female-to-male HIV transmission by approximately 60% in randomized controlled trials.^3^^-^^5^ Swaziland’s Ministry of Health (MOH) adopted VMMC for HIV prevention in 2009 with the endorsement of a national male circumcision policy and strategy. Swaziland does not have a modern-day tradition of circumcision, and the VMMC program has met with client demand challenges. Still, as of April 2014, Swaziland’s national adolescent and adult male circumcision prevalence was an estimated 24% (based on personal correspondence with Population Services International [PSI] Swaziland, which provided monitoring and evaluation support to the MOH for VMMC and early infant male circumcision [EIMC] data collection), triple the 8% circumcision prevalence reported in the 2006–2007 Demographic and Health Survey.^6^

The subsequent “Extended National Multisectoral Strategic HIV and AIDS Framework (eNSF) 2014–2018” details a rapid scale-up of VMMC to 70% coverage for adolescents and adults ages 10 to 49 by 2018.^7^ It also outlines an ambitious goal for Swaziland’s national EIMC program: 50% EIMC coverage by 2018. This strategy is aligned with a recommendation by the World Health Organization (WHO) that VMMC scale-up should consist of 2 phases: the catch-up phase and the sustainability phase.^8^^,^^9^ The catch-up phase prioritizes VMMC services for adolescents and adults, or those who are most at risk of acquiring HIV. The sustainability phase involves the progressive establishment of EIMC within the first 60 days of birth.^10^ According to modeling data from the United States Agency for International Development (USAID) Health Policy Project using the Decision-Makers’ Program Planning Tool 2.0,^11^ provision of 200,700 adolescent and adult circumcisions and 26,970 EIMCs in Swaziland would avert over 56,000 HIV infections and save US$370 million by 2035.^12^ This figure is 78% of Swaziland’s total budget for health, education, sanitation, safe water, and social protection in 2013–2014.^13^

In April 2009, with support from the United Nations Children’s Fund (UNICEF), Raleigh Fitkin Memorial (RFM) Hospital, a faith-based hospital in the commercial capital of Manzini, piloted the first EIMC model in Swaziland. The hospital introduced the pilot to strengthen delivery of maternal and newborn health services. Data from the RFM Hospital pilot demonstrated the feasibility of implementing EIMC as an integral part of maternal and newborn care services in Swaziland. Factors contributing to the successful adoption of EIMC at RFM Hospital included hospital ownership of the intervention, effective communication with stakeholders, no severe adverse events, and collaboration with development partners to meet the start-up costs of training medical staff, facility improvements, and supplies.

In October 2009, while the pilot was under way at RFM Hospital, the Swaziland MOH initiated planning for an EIMC program by hosting an international expert consultation on EIMC. Hosting the first consultation of its kind gave Swaziland a voice in the global discussion around EIMC. Swaziland’s MOH immediately moved forward on clinical and programmatic recommendations that emerged from the consultation. The resulting EIMC surgical guidelines endorsed the Mogen clamp as the preferred EIMC method for Swaziland. Less than a year after the consultative meetings, the MOH incorporated these guidelines into the existing national male circumcision surgical protocol.

**FIGURE 1:**
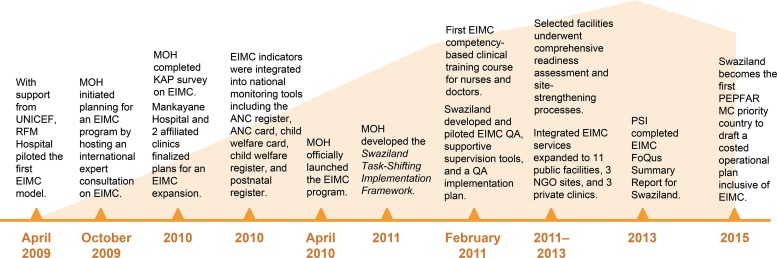
Implementation and Expansion Timeline of Swaziland’s EIMC Program Abbreviations: ANC, antenatal care; EIMC, early infant male circumcision; KAP, knowledge, attitudes, and practices; MC, male circumcision; MOH, Ministry of Health; PEPFAR, US President’s Emergency Plan for AIDS Relief; PSI, Population Services International; QA, quality assurance; RFM, Raleigh Fitkin Memorial; UNICEF, United Nations Children’s Fund.

The timeline in Figure 1 illustrates the progression of Swaziland’s EIMC program implementation and expansion. Between 2011 and 2013, through MOH leadership, funding from UNICEF and the US President’s Emergency Plan for AIDS Relief (PEPFAR), and technical assistance from implementing partners, including PSI, Jhpiego, and others, integrated EIMC services expanded to 17 facilities: 11 public facilities (referral hospitals and health centers), 3 NGO sites, and 3 private clinics. Before initiating EIMC expansion, selected facilities underwent comprehensive readiness assessment and site-strengthening processes. The site-strengthening process involved assigning space for procedures, procuring EIMC equipment and supplies, ensuring the quality of waste management and reporting systems, and training and mentoring service providers according to MOH quality assurance tools.

Between 2011 and 2013, Swaziland expanded integrated EIMC services to 17 facilities.

The MOH and stakeholders established EIMC within its national VMMC and HIV prevention program and integrated it into existing health service delivery outlets, namely the maternal, newborn, and child health (MNCH) service delivery platforms. The Swaziland EIMC experience offers a useful template for other countries with generalized HIV epidemics and low circumcision prevalence that are embarking on EIMC programming.

## METHODS

We collected data during a consultation process with both VMMC and EIMC stakeholders between March and May 2014 in preparation for the document, “Male Circumcision Strategic and Operational Plan for HIV Prevention 2014–2018.” The drafting of this important guiding document offered a timely opportunity to reflect on the first years of EIMC program implementation and consolidate lessons learned.

We collected data through structured stakeholder focus group discussions and in-depth interviews with key informants. We conducted 30 in-depth interviews with:

- Individuals from key MOH departments, including Central Medical Stores, the Public Health Unit, the Strategic Information Department, the Legal Advisor, the Swaziland National AIDS Program, the Rural Health Motivator Program, the Sexual and Reproductive Health Unit (SRHU), and the Expanded Program on Immunization (13 interviews)
- NGOs involved in EIMC service delivery and programming, including PSI, the Family Life Association of Swaziland, the Elizabeth Glaser Pediatric AIDS Foundation, and mothers2mothers (5 interviews)
- Professional associations, including the Swaziland Democratic Nurses Union, the Swaziland Nursing Council, and the Private Providers Association (3 interviews)
- Donors supporting EIMC programming (2 interviews)
- A traditional organization, Khulisa Umntfwana (“Grow a Child”)
- Providers and clinical managers working at 2 regional hospitals, 3 health centers, and 1 private clinic that offer EIMC (6 interviews with 5 doctors and 5 nurses). These were among the most informative interviews.

We collected data through structured stakeholder focus group discussions and in-depth interviews with key informants.

We selected stakeholders for in-depth interviews based on their involvement with MNCH or HIV/AIDS health issues from a policy or practice perspective, those with expertise in the Swazi health care workforce, and those with direct clinical or program experience with EIMC. While it was not feasible to visit all 17 facilities conducting EIMC services, we involved facilities from all 4 geographic regions of Swaziland as well as the varying types of facilities. Each interview lasted between 30 and 60 minutes, and we tailored them to informants’ areas of expertise: EIMC technical approach and service delivery, coordination, human resources, or communication and education.

We convened 2 focus group discussions. Traditional leaders from Manzini Region attended the first discussion, which addressed cultural values related to EIMC, and optimal approaches to building informed community demand for EIMC. The second discussion, an MNCH stakeholders’ forum with 27 participants, included 10 EIMC-trained nurses and facility matrons representing facilities across all 4 regions, as well as other NGO and national MOH representatives. Forum participants broke into small groups to discuss questions of (1) provider support and staffing for MNCH services, including EIMC, (2) EIMC entry points and service coordination and linkages between VMMC and EIMC, (3) facility-based client education and counseling, (4) EIMC demand creation and community awareness, and (5) EIMC expansion and scale-up. Note-takers documented the focus group discussions, and we analyzed the notes for consistent themes. An implementing partner responsible for VMMC and EIMC service delivery contributed all service delivery data presented here: numbers of EIMC procedures performed by month, year, and location, as well as the incidence of documented moderate and severe adverse events. All data collection and analysis were conducted according to international principles of maintaining privacy and confidentiality of personal information.

## RESULTS

With a total of 5,149 EIMCs performed between 2010 and the end of 2014, Swaziland now leads the Eastern and Southern Africa region in the scale-up of EIMC. By mid-2014, 123 health care workers (45 doctors and 78 nurses) had been trained through 11 clinical trainings. Five providers, trained as trainers, facilitated clinical trainings and conducted mentorship visits to newly trained providers. Approximately 80% of the infants were circumcised in the immediate postpartum period before discharge. The remaining approximately 20% of infants were circumcised later, and included referrals from other facilities or in tandem with immunization or routine postpartum visits. Notably, Swaziland’s EIMC program has not reported any moderate or severe adverse events to date at the time of publication of this article. Severe adverse events require extensive intervention with referral or specialist input, mild adverse events require minimal or no intervention, and moderate adverse events can be classified as neither severe nor mild, but do require intervention and are usually managed on-site. Because moderate adverse events are managed on-site, providers may have underreported these adverse events, but this did not emerge as an issue in any focus group discussions or in-depth interviews. In 2015, Swaziland became the first PEPFAR male circumcision priority country to draft a costed operational plan that includes EIMC.

80% of circumcisions took place in the immediate postpartum period.

In 2015, Swaziland became the first PEPFAR male circumcision priority country to draft a costed operational plan that includes EIMC.

### Technical Approach and Service Delivery

**FIGURE 2:**
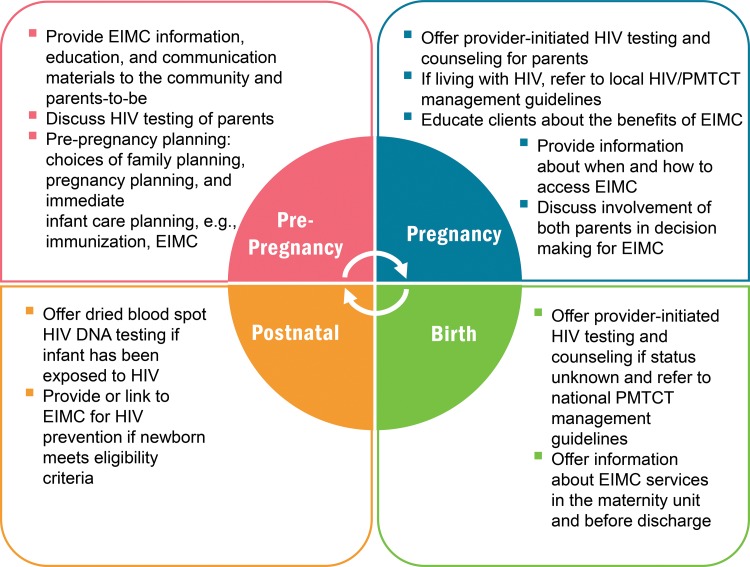
Swaziland’s Model of EIMC Integration Into Reproductive, Maternal, Newborn, and Child Health Platforms Abbreviations: EIMC, early infant male circumcision; PMTCT, prevention of mother‐to‐child transmission (of HIV).

In Swaziland, EIMC integration starts during the pre-pregnancy period and continues through the postnatal period (Figure 2). Antenatal care, labor and delivery, postnatal care—all with integrated prevention of mother-to-child transmission (PMTCT) of HIV services—as well as child welfare clinics serve as entry points for EIMC services. EIMC messages accompany those of birth preparedness, antenatal care/PMTCT, and comprehensive “day of birth” care for the mother and newborn. At the facility level, health care providers and NGO-supported EIMC motivators and “mentor mothers” offer comprehensive information and education for parents and guardians so they can make informed choices about EIMC.

Swaziland registers approximately 34,249 births per year.^14^ In the public sector, 11 hospitals and health centers across Swaziland’s 4 regions routinely provide labor and delivery services. The MOH chose these facilities for the introduction of EIMC services so that EIMC could be performed by midwives and nurses before mothers and babies are discharged. EIMC is integrated within delivery and postpartum services and offered as a routine part of the MNCH package of services for healthy baby boys regardless of HIV exposure.

In 2011, the MOH developed the “Swaziland Task-Shifting Implementation Framework in Support of Quality Health Service Provision.” According to the framework, some minor procedures were to be shifted from doctors to nurses.^15^ Although the task-shifting framework was approved, it has not been fully operationalized. However, nurses’ scopes of practice and job descriptions have been reviewed to incorporate shifted tasks, including EIMC. Currently, nurse-midwives perform EIMC in hospitals and health centers with physician backup.

According to a representative sample of both doctors and nurses at RFM Hospital, the facility with Swaziland’s most established EIMC program, when doctors and nurses are trained together, doctors feel more comfortable providing nurses the autonomy to lead the program. RFM Hospital started task shifting for EIMC in 2010 because doctors could not meet client demand for the service. Although hesitant in the beginning, hospital management embraced task shifting for EIMC because of the excellent clinical outcomes. The strong safety record to date at the time of publication of this article, as evidenced by the absence of documented severe or moderate adverse events, has further strengthened national confidence in EIMC-trained nurses. Global evidence in support of task shifting for male circumcision, particularly from Kenya, reinforces the argument in its favor.^16^

Development partners have primarily supported the procurement and distribution of EIMC instruments, including Mogen clamps, prepackaged EIMC kits, and consumables. Within the next 5 years, the MOH plans to assume responsibility for EIMC supply and equipment procurement within its supply chain systems for the MNCH platform.

In 2011, the MOH established the position of National Male Circumcision (MC) Coordinator under the Swaziland National AIDS Program. This position supports both the VMMC and EIMC programs. According to the National MC Coordinator, Swaziland’s MOH viewed the EIMC program as both a way to further its HIV prevention goals and as an opportunity to strengthen MNCH services. EIMC stands to improve health outcomes for mothers and their babies by increasing attention to improving services, including strengthening infection prevention measures, reinforcing close monitoring of newborn health, encouraging routine neonatal physical examinations, discouraging early discharge, and promoting postpartum follow-up for both mothers and babies.

**FIGURE 3:**
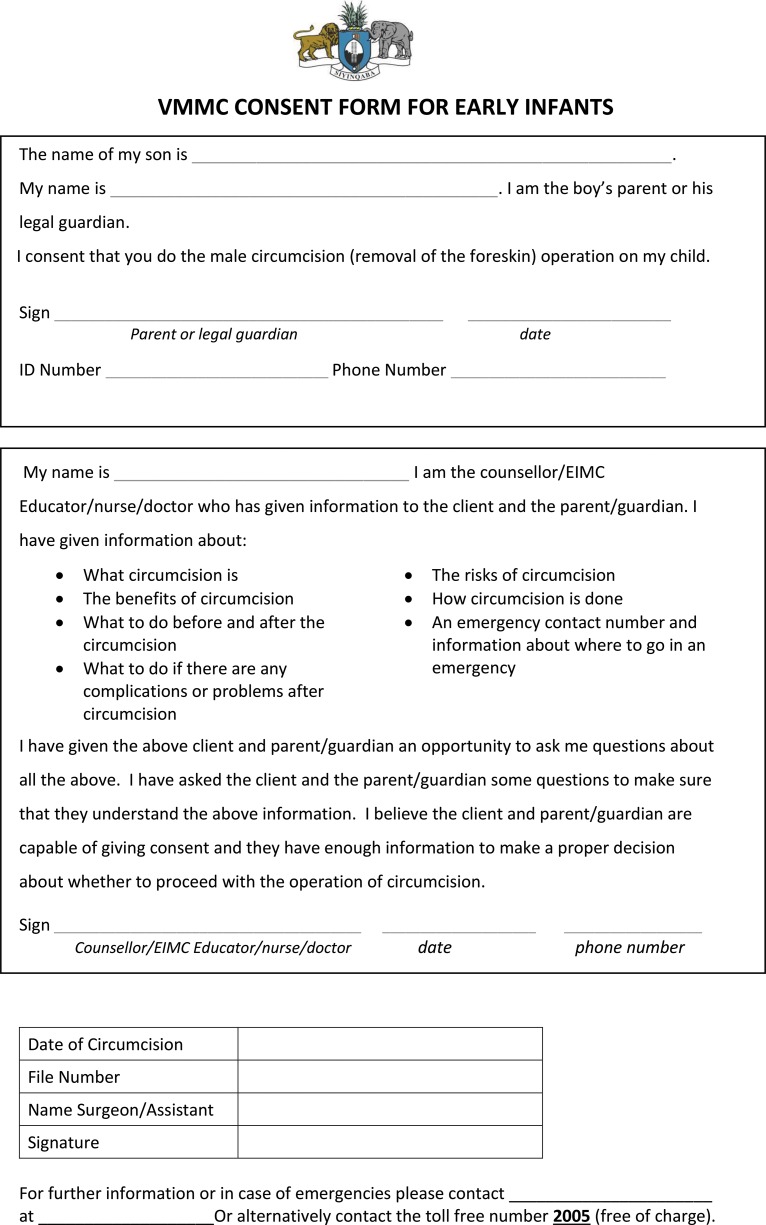
Early Infant Male Circumcision Consent Form Used in Swaziland

Swaziland has not yet completed a formal evaluation of EIMC, but EIMC integration into the MNCH platform appears to have been an effective approach to the delivery of services based on the number of EIMCs conducted as well as the following markers:

- EIMC indicators were integrated into national monitoring tools including the antenatal care (ANC) register, ANC card, child welfare card, child welfare register, and postnatal register.
- EIMC has been integrated into the comprehensive package of essential services for healthy baby boys.
- EIMC is part of the health education content discussed during routine antenatal care visits.
- EIMC consent forms for parents (Figure 3) are distributed through the MNCH platform.
- Health facility management has identified EIMC focal people.
- All public health facilities that offer labor and delivery services have allocated space to perform EIMC within their maternity units.
- Facility doctors oversee the EIMC service delivery as well as facility-level EIMC training activities.

### Coordination

In the early stages of VMMC programming in Swaziland, a technical working group led by the MOH with participation from development and implementing partners provided oversight. Under the broad technical working group for VMMC, a subgroup focused on technical aspects of EIMC. This subgroup coordinated various aspects of the EIMC program, including macro-level program planning, clinical skills training, facility identification and assessment, and quality assurance. In February 2011, this technical team developed and piloted EIMC quality assurance supportive supervision tools, as well as a quality assurance implementation plan. As EIMC expanded to additional facilities, program planners recognized that it was critical to fully engage the MNCH platform for an integrated, robust, and sustained EIMC program. Efforts are currently under way to bring the SRHU, which is responsible for the MNCH platform, fully into the coordination framework.

It is envisaged that the VMMC technical working group, under the leadership of the National MC Coordinator, will include the manager of the SRHU. EIMC issues will be addressed through ad hoc working groups under the VMMC technical working group.

### Human Resources to Support EIMC

The majority of key informants stressed that the human resource needs of an EIMC program are different from those of the intensive, time-bound, VMMC catch-up program. While EIMC is the long-term sustainability plan for VMMC, it is linked to, but also distinct from, the VMMC program. For adolescent and adult VMMC services, dedicated nurses and doctors were hired to meet the ambitious VMMC catch-up targets. MOH interviewees stated that this approach would be neither practical nor desirable for an integrated, sustainable EIMC program.

To face the human resource challenges for EIMC scale-up, the MOH, in collaboration with its partners, first trained EIMC providers, doctors, midwives, and nurses, through competency-based clinical training beginning in February 2011. Swaziland was the first country to pilot the competency-based clinical skills training in EIMC using the WHO/Jhpiego “Manual for Early Infant Male Circumcision Under Local Anaesthesia” and the associated UNICEF/Jhpiego facilitators guide and learners workbook.^17^^-^^19^ Implementing partners stated that during the planning stage, trainers realized that it might be difficult to gain informed consent for sufficient numbers of infants in order to ensure provider competency before completion of the 2-week training. In response, the MOH adapted the training program to include on-site clinical mentorship for newly trained providers until they achieved competency. EIMC-specific performance standards also reinforced best practices and post-training follow-up.

With the introduction of EIMC services, MOH stakeholders anticipated concerns about the introduction of an “additional” task without providing additional compensation. This was a particular challenge because doctors and nurses involved in the VMMC program had been compensated for the extra hours worked in support of the program. The MOH also needed to ensure that routine and emergent demands of the MNCH platform were not compromised by the introduction of EIMC in MNCH units. For these reasons, according to representatives from the Swaziland National AIDS Program, the MOH scaled up EIMC slowly. A considered, unhurried scale-up allowed time for facility teams to develop creative and practical solutions to any problems that arose. While the issue of compensation is still a sensitive one, ongoing inclusive dialogue is helpful in clarifying decision-making rationale.

A considered, unhurried scale-up allowed sufficient time for facility teams to develop creative and practical solutions to any problems that arose.

To address human resource constraints, some higher-volume sites, such as the Mbabane Government Hospital and the RFM Hospital, allocated dedicated, trained midwives to EIMC. Facility management redistributed staff despite human resource shortages and without financial incentives, which demonstrates ownership of the EIMC program at the facility level. Assistance from partner-supported EIMC motivators—to educate clients, link clients to services, and help complete paperwork—also relieved providers of some supportive functions.

### Client Education and the Informed Consent Process

Formative research has emphasized that EIMC sensitization efforts must provide parents with the information to make informed decisions.^20^^,^^21^ At present, health care providers, EIMC motivators, and mentor mothers at the facility level provide the bulk of EIMC counseling and education. Organizations, including the Elizabeth Glaser Pediatric AIDS Foundation and mothers2mothers, have been instrumental in training facility-level providers and support staff to educate clients about EIMC. At the community level, interpersonal communication agents and structured community dialogues sensitize and mobilize communities for both EIMC and VMMC. This approach provides adequate time for parents to make informed decisions or consult with extended family members. Written consent forms are available in both English and siSwati, the local language, and are thoroughly reviewed with clients (Figure 3). One parent, the mother or father, or the legal guardian, as well as the counselor or provider, must sign the consent form. Because these forms are distributed during the antenatal period, mothers have the option to take the forms home for discussion with family and partners and obtain fathers’ signatures if couples decide that they want their sons circumcised. To protect human rights, the MOH designed this rigorous consent process, supported by a national strategy that states that circumcision should be voluntary in all cases. In the case of EIMC, much like immunization, parents determine the best interests of their children.

## DISCUSSION

In reviewing EIMC program data and feedback from key stakeholders, several critical program components emerge. Distilled findings across all results areas highlight the presence of the following **factors and themes that contributed to the development** of Swaziland’s fledgling EIMC program:

- **Program backing from committed MOH leaders who have been termed early adopters in implementing an evidence-based EIMC intervention.**^22^ Despite the fact that no other country in the Eastern and Southern Africa region was implementing EIMC at the time of Swaziland’s EIMC program initiation, there was strong, early political will and commitment (via the national strategic plan) from Swaziland’s MOH.
- **Consistent and coordinated funding for EIMC across multiple funding sources during the early stages.** UNICEF funded the first EIMC pilot at RFM Hospital in 2009. Since then, PEPFAR has provided consistent funding through USAID-supported EIMC expansion, which has helped the program establish a sound foundation.
- **The role played by RFM Hospital, whose model EIMC pilot encouraged Swaziland to move quickly from EIMC adoption to program implementation.** RFM Hospital’s initiative and leadership informed the later roll-out of the EIMC program, highlighting the need for dedicated EIMC staff and space as well as linkages and coordination across the MNCH platform.
- **Targeted technical support from implementing partners.** An example of technical assistance that supported program scale-up included formative research, supported by PSI and completed in 2013, that informed EIMC program decision making. This research found that there was a near-universal perception that consent for EIMC cannot be granted by one parent alone, and emphasized that EIMC sensitization efforts must provide parents with the information needed to make informed decisions. Another example of targeted technical support included the efforts of Jhpiego’s Maternal and Child Health Integrated Program to help providers achieve competency through a mix of classroom and clinical mentorship. Other organizations, such as the Elizabeth Glaser Pediatric AIDS Foundation and mothers2mothers, also played pivotal roles in informing potential clients through community and facility education sessions.
- **Formative research that informed decision making.** A study of knowledge, attitudes, and practices (KAP) conducted by RFM Hospital helped direct the EIMC pilot and, eventually, national implementation. Later, an MOH-led, PSI-supported KAP study informed community- and facility-based EIMC demand-creation efforts.
- **A policy environment open to task shifting.** To overcome the human resource challenge, in 2011 Swaziland’s MOH developed the “Swaziland Task Shifting Implementation Framework in Support of Quality Health Service Provision.” This framework allows nurses to perform minor procedures that were previously conducted only by doctors.

While these components contributed to a growing national program, EIMC stakeholders also stated that scale-up and implementation of EIMC in Swaziland were not without the following **challenges**, listed by results area:

### Technical Approach and Service Delivery

- **Legislation is required to enact task shifting.** Although the MOH approved the task shifting framework, fully operationalizing it remains a challenge, as does securing task shifting support from all relevant stakeholders.
- **Early postpartum discharge of the mother/baby pair results in missed opportunities for EIMC.** WHO recommends that EIMC should be performed at least 12 hours after birth to ensure that babies are healthy and stable before the procedure. However, due to high birth volumes and limited space, clients are often discharged before 12 hours postpartum, despite global guidance that mothers and babies should remain at facilities for 24 hours after birth, the period of greatest risk.^23^ Addressing challenges of early discharge could both improve EIMC uptake as well as ensure better monitoring of mothers and babies after birth.

### Coordination

- **EIMC requires broad coordination across multiple stakeholders at the national level.** In Swaziland, because EIMC was conceived within the larger VMMC strategy and coordinated by the Swaziland National AIDS Program, involvement of key stakeholders from the SRHU was not as extensive as desired.
- **Program ownership should be fostered at all levels to avoid the perception of a donor-driven process.** Although the EIMC program has been implemented within public health facilities, it still depends on donor support for material and financial resources. Moving forward, Swaziland will need to assume greater financial ownership as donors limit funding for EIMC.

### Human Resources to Support EIMC

- **Human resource constraints can pose challenges for service continuity.** The EIMC program is constrained by regular staff rotations of EIMC-trained providers. EIMC services are further challenged by limited numbers of EIMC-trained providers inhigh-volume facilities and already-limited human resources for health care services.

The EIMC program is constrained by staffing shortages as well as regular staff rotations of EIMC-trained staff.

### Client Education and the Informed Consent Process

- **The consent process for EIMC is complex.** Parents should be ready to make a decision about EIMC by the time they are admitted to facilities for deliveries. Parents and key decision makers should be sensitized before pregnancy or in early pregnancy. Early education allows couples and families the time to make considered decisions about EIMC.
- **Client counseling needs dedicated educators.** Especially in the early stages of program implementation and scale-up, dedicated educators can help establish and sustain EIMC services amid the multiple demands of busy and often understaffed MNCH units.
- **EIMC has vocal global and regional opponents.** Misinformation can jeopardize fledgling EIMC programs unless these programs continue to involve and educate communities and health care workers.

## CONCLUSION

Like PMTCT, EIMC provides an opportunity for nurses to help lead the way toward an HIV-free generation. Because there are approximately 12 times as many registered nurses as doctors in Swaziland, and because an overwhelming number of public sector facilities are led by nurses,^24^ nurses and midwives have borne the brunt of the HIV/AIDS burden in Swaziland. Empowerment of nurses and midwives to take and embrace this critical role will help engender greater support for the EIMC program.

As with all VMMC programming, program staff must establish a careful balance between service delivery and community awareness. Informed demand from parents and communities for the service may also support the establishment of EIMC as a service delivery priority. However, for countries with developed VMMC programs that are now introducing EIMC for sustainability, it is also important to recognize that EIMC requires a different approach—standards and guidelines for VMMC must be expanded and adapted to address service provision on a different platform.

Swaziland has become the first country to implement EIMC programming quickly in response to the growing evidence supporting the health benefits of the procedure. In a country without a tradition of circumcision, where the sociocultural norms are not favorably predisposed to EIMC, and where VMMC scale-up faced challenges related to client demand, the EIMC program has expanded, which is particularly impressive because of the absence of a targeted national EIMC communication campaign. Continued expansion holds the promise of averting thousands of HIV infections and is a large step toward creating an HIV-free generation in Swaziland.

## Notes

**Peer Reviewed**
